# Changing a Community: A Holistic View of the Fundamental Human Needs and Their Public Health Impacts

**DOI:** 10.7759/cureus.44023

**Published:** 2023-08-24

**Authors:** Uzochukwu Adabanya, Ayoola Awosika, Jin Hyung Moon, Yenamala Ushasri Reddy, Ferdinand Ugwuja

**Affiliations:** 1 Community Medicine, Mercer University School of Medicine, Columbus, USA; 2 College of Medicine, University of Illinois, Chicago, USA; 3 General Medicine, Mercer University School of Medicine, Columbus, USA; 4 Hematology, Rush University Medical Center, Chicago, USA

**Keywords:** health promotion, community collective power, public health and safety, gentrification, community engagement

## Abstract

There are many approaches to changing a community to ensure it serves the people’s fundamental needs. For example, enabling equitable access to critical aspects of the community, such as quality healthcare, high-quality education, and job training, is vital for promoting community safety through enhancing tolerance and respect for diversity. However, creating a community that serves the fundamental needs of the people demands a substantive investment of effort. Understanding the nature of these efforts requires discussion of community engagement, examining community networks and their role in fostering cooperative action, enhancing public safety, and identifying the structures of involvement and pertinent routes for developing community land. Understanding such efforts entails knowing the issues related to gentrification and disbandment. These hands-on possibilities can help avert the possibility of people being pushed out of their community settings. These insights further shed light on how the family unit and larger community are able to create collective unity and foster each member’s responsibility in community service provision that promotes community integration. Examining how violence and other factors affect a community’s collective power is necessary to determine how a community can avoid such violence and encourage positive changes at the individual and family levels to promote community cooperation and safety. Essentially, changing a community can yield significant improvements in public health. Addressing factors such as access to nutritious food, healthcare, physical activity, and social amenities and fostering social cohesion through community engagement can collectively contribute to reducing the burden of chronic diseases and promoting overall well-being. This review provides insight into crucial issues that have long plagued the societal disconnect between the local community and the leadership, policymakers, or other authoritative institutions that govern them, thus affecting the implementation of strategic social and public health initiatives. We will also explore strategies to mitigate these potential pitfalls.

## Introduction and background

**Figure 1:**
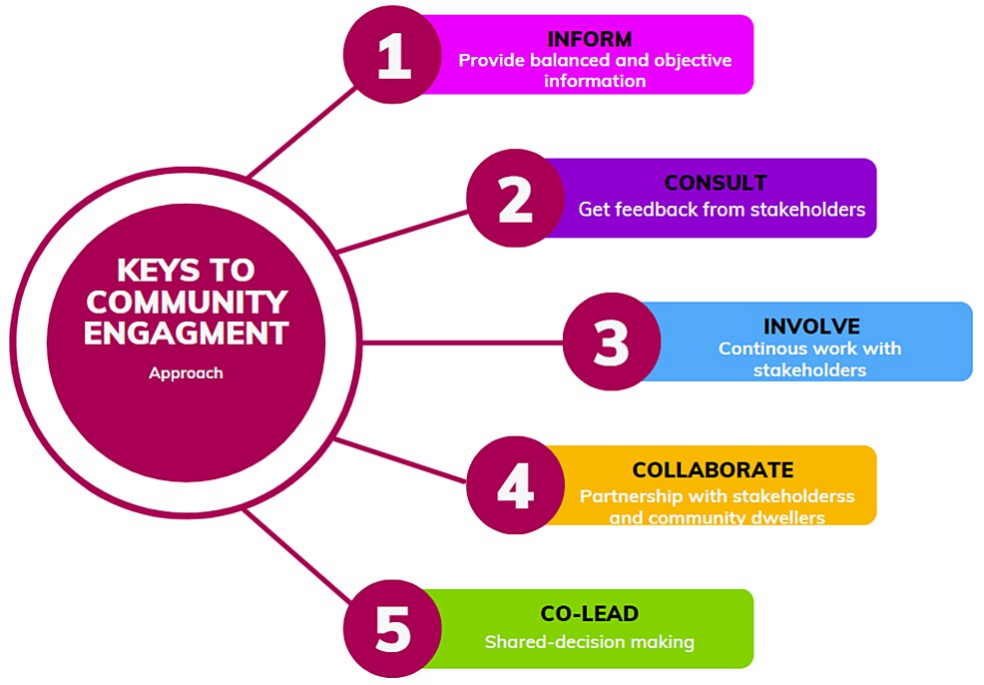
Approach to fostering community engagement. This is an original image designed by the authors using Canva design

Creating a community that serves the fundamental needs of the people demands a substantive investment of effort. Understanding the nature of these efforts requires a discussion of community engagement. Public health initiatives guided by community insights are more likely to result in sustainable changes and improved health outcomes [1]. Community engagement is a process whereby people, organizations, and groups dialogue with the public to inform their decisions on concerns that affect them. The process focuses on listening to the community and collecting information about people's wants. It also involves respecting leadership and other authoritative institutions that govern them [1]. The information is collected by asking questions and inviting discourse through various forms of established communication platforms, such as social media, that are applicable in the contemporary era. These observations imply that community engagement is a process that builds respect, trust, and understanding between the community and the organization or group, as represented in Figure 1.

It is an ongoing process that involves developing relationships with people within the community setting to understand their needs by inviting their insights through forums such as interviews, surveys, or focus groups to help create awareness about potential approaches to address the issues at hand. Such understanding enables the involved groups or organizations to develop approaches such as services or programs that resonate with the concerned community’s needs [2].

Consequently, community engagement allows community members to obtain accurate data about issues affecting their environment. According to Alter et al. [1], community engagement ensures that the people within a community setting are part of the remedial process and have a say in what happens. This participation also ascertains their understanding of the situation and how to get involved if they so desire. There are numerous vital views on community engagement, all of which can help to shape how communities work together. One such view is the concept of asset-based community development. This view focuses on the resources and strengths within a community rather than examining what is lacking. This perspective underscores working with what is already there to engender positive change [3]. Another vital view of community engagement involves focusing on issues in the social justice domain. Social justice strategies deal with power imbalances and systemic inequalities within a community. This work usually involves challenging prevailing narratives and promoting marginalized voices [4]. Community engagement can be a controversial topic and involves various challenges; however, it is fundamental for creating lasting change.

There are numerous reasons to support the benefits of community engagement. For instance, community engagement is vital to building lasting community networks, fostering cooperative action, and enhancing public safety. Weger (2018) advocates that robust community engagement is a critical component of a person’s health and a platform for advancing new collaborative care models [5]. Community engagement engenders social cohesion and trust that are fundamental for effective communication and collaboration among community members, which creates an environment that enables both individual and community progress. Moreover, community engagement allows people within a community setting to share resources and information, identify common goals and interests, and develop shared solutions to issues impeding their safety, progress, and well-being [6].

Numerous different approaches to community engagement exist. However, they all share the common goal of empowering groups and individuals to work collaboratively to improve their communities. Participation is important because it can be applied to authenticating the external approach and can be integrated into social and community development projects to render them more cost-effective [7]. Some popular engagement approaches include community-based research projects, arts-based programs, youth empowerment agendas, participatory mapping, and citizen science initiatives. Regardless of the engagement approach employed, community members must feel ownership over the process to guarantee its success. While community engagement delivers varied vital benefits, it also has several challenges. For example, it is challenging to determine the approaches to engaging with populations that are hard to reach, such as those with limited English proficiency or the economically disadvantaged. For example, it is challenging for some communities to discuss issues concerning crime or race within specific community settings, because of the risk of being politically incorrect, which could upset community members. It may also be challenging to sustain a long-term commitment when funding sources dry up or people’s lives are already busy. However, these challenges cannot undermine the critical role of community engagement in building strong communities [8].

Community engagement is, therefore, beneficial for encouraging the formation of community networks that engender cooperative action among community members, encouraging collaborative problem-sharing and resource exploitation for individual and community progress [2, 3, 7]. Such enhanced community cooperation plays a significant role in enhancing public safety, promoting social cohesiveness, and even averting issues such as gentrification that push people out of their communities and expose them to detrimental impacts such as poor socioeconomic status characterized by homelessness, joblessness, and poverty. Besides, community cooperation resulting from enhanced engagement provides community members with political capital that enables them to influence the relevant political machinery to engage in initiatives that benefit the community. Overall, changing a community can have a substantial impact on improving public health policies and health outcomes. This review will provide insight into crucial issues that have long plagued the societal disconnect between the local community and the leadership, policymakers, or other authoritative institutions that govern them, thus affecting the implementation of strategic social and public health initiatives. We will also explore strategies to mitigate these potential pitfalls.

## Review

Community networks, cooperative action, and public safety

Several theoretical frameworks underpin the role of public health in community engagement, such as the Social Ecological Model and Community-Based Participatory Research (CBPR). These frameworks emphasize the interconnectedness of individual, interpersonal, community, and societal factors that influence health behaviors and outcomes. Community-Based Participatory Research, in particular, emphasizes collaboration between researchers and community members in all stages of the research process, fostering a sense of ownership and empowerment.

Community networks are a fundamental part of this framework. They provide a way for community members to connect and share knowledge and resources that can help keep everyone safe. For example, in the contemporary era, community networks can play a critical role in connecting underserved populations in community settings to internet connectivity [9]. Community networks allow for cooperative action among a community’s members that enables them to address safety concerns and enhance communication in the event of an emergency. Numerous forms of community networks exist; some are based on shared values or interests, while others are based on geographic location. Community networks are. therefore, a form of social network with a shared goal, purpose, or interest that connects members [10]. These networks can be applied for various purposes, including cooperative action and public safety.

Cooperative action is an essential component in making community networks function correctly. Generally, a community is a group of people inhabiting the same local geographical area or with an element of shared social identity [11]. Community networks function by working collaboratively to solve problems, offering assistance and support to community members, and sharing resources. Working together enables members of a community to pool their capacities and resources to resolve issues affecting them. Cooperative action in a community setting involves any joint effort that allows community members to accomplish complex tasks or those impossible to achieve when working alone. For instance, community members could cooperate to deliver initiatives that benefit the community, such as planting trees, building a playground, or cleaning up a park. Community networks provide resources and support that can help keep people within a community safe.

Community networks support cooperative action that, in turn, boosts public safety. Public safety is, therefore, a common target of cooperative action because working together enables community members to keep each other safe from accidents and crime. For example, they might install streetlights, organize neighborhood watch groups, or provide first aid training. Public safety is an important consideration when establishing any type of network within a community setting because community engagement is a fundamental constituent of the local crime prevention program that can only function with enhanced public awareness about the existing safety initiatives [12]. Essentially, a community should have established approaches to communicating during emergencies, dealing with actual and potential threats, and enhancing community members’ safety. Public safety enhanced by cooperative action allows community members to commit public safety officials to serve their role of keeping communities safe by enforcing the appropriate laws.

The synergism of cooperative action, community networks, and public safety are conjoined and are all essential elements in creating a safe and flourishing community. By working collaboratively, residents within a community setting can identify potential problems and proactively take steps to prevent them from occurring. Community safety, therefore, depends on members within a community establishing and effectively exploiting robust networks to safeguard their resources, such as community land, public parks, and other vital resources, and improve their safety and well-being. Community residents can identify problems and develop solutions that make their neighborhoods more habitable by working together. Through community engagement, residents also build relationships with local law enforcement, creating a sense of trust and cooperation and avoiding issues such as disbandment that create a fertile platform for destructive issues such as the disbandment of critical community service-providing organizations.

By coming together to address issues such as poor housing conditions, poverty, unemployment, and lack of access to healthcare, social services, and education, residents can create sustainable change that encourages community uptake of important initiatives such as enforcing environmental sustainability practices from companies and groups within their settings and committing community-based agencies to practices that are sensitive to climate change concerns. Crime rates and detrimental events such as illicit drug use and school dropout incidences decline when people feel invested in their community. When people in a community work together towards a shared goal, they develop a sense of pride in their community. This can foster increased civic participation, such as volunteering or voting. Furthermore, robust community networks and cooperative action enhance social cohesion, which increases informal surveillance by members of the community.

Structures of involvement: the routes for developing community land

Structures of involvement are the self-help, informal, and formal networks that conservationists can employ to build community support for protected land. Informal structures comprise individual relationships and mutual aid among persons, families, and communities. Formal structures entail independent environmental groups, nonprofit organizations, or government agencies. Self-help networks feature voluntary groups of individuals who develop their own approaches to accomplish common goals. Structures of involvement, therefore, refer to the varied routes people can employ when developing a community land expectation, and they comprise individual, collective, and institutional participation [13].

Involvement structures, therefore, provide a way for communities to come together and work towards a common goal. A community must understand that each route has an array of drawbacks and benefits that need consideration before determining which one meets its needs. Consequently, the most effective approach depends on the specific resources and needs of the community in question. The most common involvement structures for developing community land trusts typically fall into one of three categories: grassroots, government, or hybrid [14]. A community land trust (CLT) is a nonprofit organization that creates and stewards commercial spaces, affordable housing, community gardens, and other critical real estate for the local community’s benefit. A community land trust’s mission is to ensure that these assets are permanently accessible to and affordable for moderate- and low-income people and to provide opportunities for neighborhood revitalization and economic development [15].

Despite using various structures of involvement, all community land trusts share a joint commitment to featuring as many stakeholders as possible to ensure that decisions are made democratically and for the benefit of the people most affected. This usually includes holding public meetings where members can express their concerns and provide input on proposed policies or projects. The grassroots approach is the most common route, and it involves a group of people coming together to form a community land trust [16]. This is achievable through an existing organization or by creating a new organization purposely for creating a community land trust. Grassroots organizations are usually the most effective at community mobilization and getting people in the community to participate in the development process [15, 16]. However, these grassroots organizations can also be the least stable because they depend on donations and volunteer labor. It also means working closely with associates, such as businesses or local government agencies, to ensure everyone is on board with what is being proposed.

The participation of so many varied entities can sometimes slow down decision-making, for instance, due to possible disagreements between the parties. However, this involvement structure also guarantees that no one group wields excessive power over what happens with community land trusts, which is fundamental to upholding long-term affordability for commercial spaces or housing developments within them [17]. The government route characteristically involves a degree of government support that comes in the form of technical support, financial assistance, or both. Government agencies can provide funding and stability, although they may not be as responsive to community needs or as committed to the project’s success. The hybrid approach combines the government and grassroots routes and generally includes a degree of partnership between the two. The hybrid route can also involve the participation of private developers.

Developing community land expectations involves identifying the structures through which individuals are involved in community land development, such as governments or informal and formal organizations. Once identified, looking for approaches to strengthen these structures and enhance their participation in land development is vital. For instance, it may help to promote the formation of new organizations or membership within existing organizations. Alternatively, it may help to participate with businesses to develop practices and tools that support community land use. These interventions have the potential to address various determinants of health, creating an environment that fosters healthier lifestyles and reduces health disparities.

Gentrification, disbandment, and hands-on possibilities

The combination of gentrification, disbandment, and hands-on possibilities can profoundly impact a community. Gentrification entails renovating and improving a neighborhood generally considered run-down or poor [18]. Gentrification involves improving a neighborhood to make it conform to middle-class taste and is usually accompanied by the displacement of residents with low incomes with the increased influx of higher-income families and new businesses. The concept of gentrification has several aspects that might be of advantage to certain areas. For instance, increased property values make an area desirable for developers and investors, bringing investments into neglected areas that can generate more job opportunities. Gentrification also has negative impacts, such as the displacement of low-income people. Government investments and policies can play a role in gentrification by incentivizing redevelopment projects and providing tax breaks.

However, gentrification may cause the disbandment of the businesses and local organizations that serve these residents. Disbandment, therefore, occurs when an organization that has been providing support or services can no longer do so, leaving those who relied on it struggling to find other sources of support and help. Disbandment, therefore, leads to the forced displacement of inhabitants who have been living in an area for a long time [19]. This outcome can cause financial hardship and even cause homelessness as people within the affected community are compelled to move to new areas where housing is more costly. Moreover, it can lead to the loss of community ties and support systems that the affected people have come to rely on.

This can also engender feelings of betrayal and anger in those displaced because they see their neighborhoods and homes being taken over by newcomers with minimal regard for their history or well-being. Further, the disbandment of local institutions and organizations can negatively impact the community, as these institutions and other relevant groups provide essential services and support to residents [20]. Without these services and support, residents may struggle to access basic needs such as healthcare and food. The loss of these institutions can engender a sense of exclusion and isolation from the larger community. This outcome can lead to higher crime rates as people feel detached from society and turn to unlawful activities to meet their needs. Ultimately, the disbandment of local institutions due to gentrification can have several adverse outcomes for residents, rendering it difficult for them to flourish in their new environment.

However, there are numerous hands-on possibilities for community members to participate in the gentrification process to ensure the process benefits everyone in the neighborhood. Hands-on possibilities can help to prevent gentrification by ensuring that people are not displaced from their neighborhoods or homes because they cannot afford to stay in them. Hands-on possibilities combat gentrification by offering people in a community setting opportunities to get involved in the community in various ways. For example, a community can engage community developers to plan equitable development projects to promote equal access to critical infrastructure in the community [21]. Another necessary hands-on approach is promoting and supporting the growth of local businesses, particularly those owned by women and people of color. These initiatives can help create jobs and economic opportunities for residents at the risk of displacement by gentrification. Further, working with existing residents to chronicle their experiences and stories can help raise awareness about the issue and foster solidarity within the community. Another approach is creating community land trusts that help keep housing affordable for low-income residents. Further, residents can work with city officials to amend zoning laws to help prevent new developments from pricing out existing inhabitants. Finally, organizing the community to participate in the political process can guarantee that decisions are made considering all residents’ needs.

Family unit and community: an intervention to create a collective unity

Family is society’s basic unit of support and advocacy. It is the basis upon which social stability and order are built [22]. The family unit is how people in a community learn to love, trust, be trusted, be cared for, and care for others. Family teaches individuals in a community how to communicate with one another and steer the world around them. Therefore, family is the first teacher in life for every individual because of its defining role in shaping the earliest experiences as well as influencing them into adulthood. These observations support the idea that a family unit is the community’s most essential and fundamental building block of life. A healthy and strong family unit creates a robust foundation for a flourishing and resilient community, not only from a sociological aspect but also from a healthcare perspective [22]. Consequently, united families can work together to engender positive change in their community. By working collaboratively, families can create interpersonal relationships with other familial units, along with the institutions and services that serve their community. These crucial relationships can create a sense of unity within the community that is powerful in creating long-term change while imprinting change on generations to come [22].

Establishing collective unity within families and communities is the intervention that engenders the desired progress at multiple societal levels. Collective unity implies joint solidarity for everyday purposes and working cooperatively toward shared goals. Congruently, families and communities should emphasize integrating additional support for each other during hardships, which would increase their resilience against challenges [23]. Consequently, collective unity in families and communities is developed through improving relationship quality through collaboration and communication between individuals and groups. Another method to improve collective unity is by generating and disseminating educational initiatives that instruct individuals within a community setting about their particular roles, responsibilities, and rights as members of society. These initiatives can educate individuals by providing them with knowledge on how they contribute to society on a much larger scale, as well as how their actions can adversely or desirably impact society overall.

Consequently, collective unity begins at the individual level and transcends into the family level and, ultimately, into the community level. This observation suggests that collective unity takes effort, patience, and time from each individual within a family and community setting. Furthermore, collective unity within a community setting creates a sense of community spirit and belonging. When individuals feel like they belong to a community, they are more likely to be engaged and invested in its success. Moreover, collective unity can help produce social capital within a community setting, which is essential for a prospering and thriving community [24]. Collective unity also promotes cooperation and collaboration among community members, leading to significant innovation and increased creativity. Lastly, collective unity can help engender an environment where individuals feel empowered, protected, and supported, which is critical for a healthy community at a spiritual, mental, and physical level. By shedding light on the family unit, the community can communicate more efficiently, forming a solidarity that can be utilized to improve public health. A sense of belonging and strong social networks have been linked to positive health outcomes, including reduced stress levels and improved mental well-being [25].

Citizens’ responsibilities in community service provision

Citizens serve varied responsibilities germane to community service and indispensable for community socioeconomic progression. An individual’s energetic involvement in a community reflects their civic responsibility to society. [26] In light of this, in some instances, the law mandates specific forms of community service, such as military service and jury duty, for community members. In other instances, locals could donate their time and money to help improve their communities. Likewise, nonprofits or the government could also engage citizens to offer community services. Despite the individual’s form of community service, participation in this element demands certain expectations and responsibilities. For instance, citizens participating in community service must commit to making positive differences in their communities. Such commitment manifests through hard work by uplifting and investing their intellectual capacities in order to mobilize the support required to fulfill their responsibilities and mandates. Furthermore, those engaged in community service must demonstrate competency by being disciplined, punctual, and dependable in order to execute their defined responsibilities.

Moreover, citizens serving various responsibilities in the community should act with a spirit of cooperation and teamwork by acknowledging their role [27]. Such collaboration contributes to enhancing the outcomes of community service for families and the general community.

Citizens providing community service are mandated to understand the needs of the community and to avail of services in order to meet those needs. They must realize that their role is not only restricted to service provision but also in the decision-making process and advocating for community betterment, a reflection of civic accountability. Furthermore, citizens in community service have a responsibility to participate actively in local government elections, particularly at the ward level. This can directly influence decisions on problems affecting them as a community, such as education, security, and development, by supporting law and policy formulation [28]. Therefore, citizens in community service must actively participate in local government decisions that concern the community by addressing central issues affecting the community’s resources. These concerns can be addressed by writing letters to elected personnel or attending city council meetings. They must participate in elections to help choose leaders who will support the protection of community resources. The observation indicates that citizens in community service are responsible for protecting community resources by preventing the destruction or loss of community assets. Likewise, citizens are responsible for promoting community assets through renovation, repair, and enhancement. They must safeguard public health by minimizing and preventing pollution of water, air, land, or other natural resources. Citizens must also encourage the formation and effective implementation of policies and programs that dispose of waste responsibly, such as recycling initiatives that manage household and commercial garbage collection.

Furthermore, they should advocate for the abatement of all identified environmental hazards that threaten their community resources and overall well-being. Citizens should engage in accountability in their community service by notifying relevant authorities when an existing operation is altered, misused, or terminated. Additionally, it is important to notify the community when a specific project within the community requires a permit, such as the removal of the existing landscape, mounting a new landscape on private property, altering any structure on private property, or demolishing any property. It is vital for the efforts of community service to protect community resources at all costs. The community can perform more effectively with the improvement of communication and collaboration through efforts to cease vandalism and prevent illegal activities. The benefits and rewards associated with participation in community service and the execution of responsibilities are immense. For instance, they protect community resources and support systems that, in turn, create a healthy and safe environment for everyone. Their actions protect the rights and well-being of several populations, such as children, women, and youth. In addition, many other vulnerable populations are also protected, such as the elderly and those with disabilities, which ultimately leads to the promotion of community cohesiveness.

Escaping from violence and changes in the community’s collective power: an insight into the causes and outcomes of changing a community

Numerous factors drive individuals to escape from their community. These factors can negatively affect a community’s capacity to form integrated community networks, which are critical for protecting community resources and promoting overall welfare for the community and the general public. Violence negatively influences individuals to leave a community, and the escape manifests in several forms. For instance, some individuals leave the community and never return, while others stay in the community but never leave their homes for years. Some individuals stay hidden, while others escape into a life of crime or drug addiction as a way to cope and protect themselves. Regardless of the approach that they choose to partake in to escape community violence, the individuals affected by violence seem to never participate in any meaningful community engagements, consequently draining the community’s collective power [29]. Such withdrawal from the community setting results in various changes to the community’s overall fabric. This continually undermines a community’s ability to protect or effectively exploit its networks for the advancement and protection of the community as a whole.

Aside from violence, economic development is one of the major catalysts for change in a community. Such development can occur when new businesses move into the area and offer higher-paying jobs, which causes residents to relocate in search of better opportunities. This will often lead to gentrification, where wealthier populations displace lower-income residents who can no longer afford the constant increase in rent and property value. Although this can lead to positive outcomes like improved infrastructure and safety, it also has negative consequences like increased inequality and the displacement of long-time residents. A desire for social change within a community can also engender transformation within the community. For example, individuals may shift from a community if they suspect that their environment lacks the necessary resources. This internally leads to lessened community productivity, a weakened community social fabric, and reduced overall community capacity that would have mobilized its resources in order to access desirable change.

Many factors can contribute to changes in a community’s collective power. For example, a desire for social justice shifts populations to advocate for positive change in the community. Citizen-led accountability engagements can solidify collective efforts to address health inequality issues in a community system and foster better health system governance [30]. Another important motive is a shift in demographics. For example, a population’s composition or size change within a community structure can significantly impact the community’s overall power. For instance, if there is an influx of new residents within a community setting, this population may not be as invested in the community, its institutions, and overall welfare as those who have been living there for generations. Such a change in community welfare commitment can stimulate a decline in civic engagement and participation, which deteriorates the community’s collective power. Economic recession or growth is another important factor that can significantly alter the collective power within a community. During economic adversity, community members are less likely to donate financial resources as well as time to other causes outside of their immediate needs. This portrays how reduced resources at the community level can decrease a community’s collective power. Conversely, when individuals feel more financially secure, they are more likely to get involved with philanthropic activities and support their local community through monetary contributions or volunteerism. Therefore, it is critical to support the community’s collective power in allocating the necessary resources for progression. A change in leadership can cause a transition in a community’s shared capacity as well as change the community’s economic status or demographics.

An admirable leader can enhance a community’s collective power by facilitating the implementation of favorable policies. Outside forces, such as the media or government authorities, can also impact the community’s power dynamics. Ultimately, it is essential to remember that collective power is always shifting and evolving, and there are many complex factors at play. It is important to note a few disadvantages of undesirable change in a community’s collective power that can manifest. Particularly, this can lead to instability and conflict, mainly due to the resulting possibility of an uneven distribution of resources that can further aggravate tensions within a community. Collective power changes in a community can also instigate the loss of traditional customs or knowledge as new members occupy positions of authority, potentially diluting social cohesiveness. However, positive changes in a community’s collective power can avail several advantages. For instance, improvement in leadership can give community members a sense of shared purpose that is necessary for facilitating resource mobilization and alleviating issues such as poverty, homelessness, and other factors that lead to low socioeconomic status. Likewise, it is important to note that positive changes can also propel socioeconomic advancement at the community level [10, 11].

## Conclusions

Changing a community can have a substantial impact on improving various public health outcomes. This research sheds light on the intricate relationship between fundamental human needs and their profound impact on public health within a community context. By adopting a holistic approach that encompasses community engagement, network building, cooperative action, and other key factors, we recognize the significant potential to drive positive change. 

The evidence presented underscores the vital role of community engagement and networks in fostering social cohesion and emotional well-being. When communities come together to address health, social, or infrastructural concerns, it leads to collective efforts that result in sustainable changes. For instance, grassroots campaigns advocating for smoke-free public spaces can contribute to reducing smoking rates and exposure to secondhand smoke. Collaborative action within communities contributes to improved public safety, disaster preparedness, and crime reduction. However, the challenge of gentrification and disbandment highlights the need for sensitive urban planning to mitigate adverse effects on vulnerable populations and maintain the fabric of community life.

Empowering communities with collective strength and advocating for better public health policies proves essential for driving positive health outcomes. Effective health planning and promotions, not only address specific health needs but also work towards reducing health disparities among diverse socioeconomic groups within a community. As we move forward, it is imperative for policymakers, community leaders, public health practitioners, and researchers to embrace these findings. By recognizing the symbiotic relationship between fundamental human needs and community health, we can facilitate targeted interventions that enhance overall well-being and promote health equity. The call for further research in this area invites exploration into the long-term effects of community interventions and the evolving role of technology in community well-being.

In essence, this research underscores that changing a community's trajectory towards improved public health necessitates a comprehensive understanding of fundamental human needs and their intricate interplay. Through collaborative efforts, informed policy-making, and a commitment to inclusivity, we can pave the way for healthier, more resilient, and thriving communities.
